# Supplementary material for: Forecasting the spread of SARS-CoV-2 is inherently ambiguous given the current state of virus research
Source: PLoS One. 2021 Mar 3;16(3):e0245519. doi: 10.1371/journal.pone.0245519 (PMC7928451; doi:10.1371/journal.pone.0245519)
Supplement: S1 File — (ZIP) [file pone.0245519.s001.zip › SupportingInformation.pdf]

S1 Appendix. Case fatality ratio

The case fatality ratio, i.e. the probability that an infection leads to death, was estimated following three different approaches. The case fatality ratios used in scenario "Data from NL" were defined as the estimated death toll in the Netherlands divided by the estimated number of infected individuals. We assumed that this provides the probability that someone who gets infected, dies from COVID-19 ($P(\texttt{D}|\texttt{E})$). In scenarios Literature-E and Literature-I-s we used case fatality ratios obtained from the literature [30], where we assumed that these ratios reflect $P(\texttt{D}|\texttt{E})$ for Literature-E and $P(\texttt{D}|\texttt{I-s})$ for Literature-I-s. The computed case fatality ratios for each scenario are provided in Table 1.

Age group	Data from NL ($P(\mathrm{D}|\texttt{I-s})$)	Literature-E ($P(\texttt{D}|\texttt{I-s})$)	Literature-I-s ($P(\texttt{D}|\texttt{I-a})$)
0-9	0	0	0
10-19	3.32×10^{-5}	2.00×10^{-3}	2.00×10^{-3}
20-29	4.00×10^{-5}	2.00×10^{-3}	2.00×10^{-3}
30-39	8.13×10^{-5}	2.00×10^{-3}	2.00×10^{-3}
40-49	1.62×10^{-4}	4.00×10^{-3}	4.00×10^{-3}
50-59	1.03×10^{-3}	1.30×10^{-2}	1.30×10^{-2}
60-69	6.43×10^{-3}	3.60×10^{-2}	3.60×10^{-2}
70-79	4.82×10^{-2}	8.00×10^{-2}	8.00×10^{-2}
80+	2.43×10^{-1}	1.48×10^{-1}	1.48×10^{-1}

Table 1: Case fatality ratios used for the three different CFR scenarios.

For scenario "Data from NL" we needed estimates on the number of deaths and the number of infected individuals. The death toll as reported by the Dutch national health institute, RIVM [27], is likely an underestimation: many deaths caused by COVID-19 may not be reported as such because COVID-19 was not confirmed or even suspected. Another estimate for the number of COVID-19 deaths can be obtained from the excess deaths in 2020 compared to 2015-2019 based on weekly death rates reported by Statistics Netherlands [28]. Since Sanquin reported test results on blood samples obtained up to and including week 15, we used the excess deaths up to and including week 16 for computing the CFR. The extra week was to account for the time from infection till death. CFRs were then computed based on the average between the death toll reported by RIVM and the excess deaths reported by Statistics Netherlands. Details on the number of deaths reported by RIVM, excess deaths and the average between the two are provided in Table 2. Note that no excess deaths were observed for people under 70 years old.

The number of infected individuals was estimated based on research by Sanquin, the Dutch blood bank, who tested all blood donations of individuals between 20 and 60 years old for antibodies and found that on average 3% of donors has developed antibodies [29] up to 13 April. Estimates for age groups 0-9 and 10-19 were obtained from the Pienter investigation [29]. People over the age of 70 rarely donate blood, and no estimates of

the infected fractions were available. We therefore fit a linear relationship between the number of daily contacts and the infected fraction for each age group, and used this to estimate the fraction of infected individuals for all age groups above 20 years of age. Details on the final percentages used for computing the CFR are provided in Table 2.

Table 2: COVID-19 deaths as reported by RIVM, excess deaths as reported by Statistics Netherlands and the average of the two up to and including week 12. Excess deaths were only observed for the age groups 70-79 and 80+.

Age group	Death rates			% of the population infected	
	COVID-19	Excess deaths	Average	Sanquin/Pienter	Estimated CFR
0-9	0	-	0	1	1
10-19	1	-	1	1.5	1.5
20-29	3	-	3	3.6	3.4
30-30	6	-	6	3.4	3.5
40-49	13	-	13	3.5	3.5
50-59	87	-	87	3.1	3.4
60-69	332	-	332	2.8	2.5
70-79	1084	1274	1179		1.6
80-150	1636	4277	2957		1.5

S2 Appendix. Actual number of deaths

The death toll reported by RIVM is an underestimation on the real death toll. RIVM reported a total of 602 COVID-19 induced deaths up to March 25 and we used this as a lower bound in our verification of parameter combinations; if the number of deaths were not at least equal to 602, we knew that the parameter combination was not realistic. An upper bound can be obtained from the excess deaths reported by Statistics Netherlands in comparison with the years 2015-2019 [28]. However, we computed the excess deaths not only by comparing the number of deaths in a week to the number of deaths in the same week in 2015-2019, but also to the number of deaths occurring in previous weeks in 2020. Thus the upper bound on the number of deaths in each week of our study is computed by taking the maximum difference between the reported number of deaths by CBS in this week and the reported number of deaths in this particular week in the years 2015-2019 and the number of deaths reported in week 1 up to (but not including) this week in 2020. This gives us an upper bound of 2139 up to and including March 25, and in case the simulation has more deaths than 2139 the parameter combination used is considered unrealistic.

Scenarios			Transmission probability								
Symptoms	CFR	Immunity	0.15	0.2	0.25	0.3	0.35	0.4	0.45	0.5	0.55
37.5%	Data from NL	All	280	383	561	822	1205	1799	2572	3576	4859
37.5%	Data from NL	High	276	379	551	821	1235	1869	2758	3897	5292
37.5%	Data from NL	Medium	281	378	556	838	1248	1873	2764	3887	5335
37.5%	Data from NL	Low	271	368	534	819	1228	1879	2749	3906	5304
37.5%	Literature-E	All	306	439	647	993	1506	2253	3239	4508	6089
37.5%	Literature-E	High	321	453	661	1008	1532	2304	3381	4780	6486
37.5%	Literature-E	Medium	319	450	678	1007	1523	2297	3343	4756	6550
37.5%	Literature-E	Low	327	457	671	1015	1547	2335	3377	4812	6595
37.5%	Literature-I_s	All	310	450	690	1059	1630	2489	3763	5511	7865
37.5%	Literature-I_s	High	323	468	687	1061	1647	2513	3821	5610	8043
37.5%	Literature-I_s	Medium	312	456	683	1037	1622	2500	3820	5692	8127
37.5%	Literature-I_s	Low	319	454	668	1019	1591	2474	3763	5616	8082
50.0%	Data from NL	All	336	498	783	1231	1940	2972	4364	6127	8141
50.0%	Data from NL	High	342	506	789	1251	1988	3048	4562	6474	8734
50.0%	Data from NL	Medium	339	518	821	1379	2141	3386	4986	7047	9557
50.0%	Data from NL	Low	342	520	822	1325	2119	3276	4906	6951	9392
50.0%	Literature-E	All	413	667	1099	1806	2938	4494	6617	9265	12353
50.0%	Literature-E	High	424	668	1137	1858	3027	4698	6946	9813	13248
50.0%	Literature-E	Medium	422	659	1116	1838	3047	4712	7030	9936	13364
50.0%	Literature-E	Low	430	666	1125	1828	2996	4661	6923	9764	13226
50.0%	Literature-I_s	All	416	674	1138	1959	3212	5175	7928	11624	16109
50.0%	Literature-I_s	High	433	696	1156	1963	3265	5290	8062	11966	16835
50.0%	Literature-I_s	Medium	424	697	1168	1988	3332	5399	8297	12118	17162
50.0%	Literature-I_s	Low	431	696	1173	1978	3307	5288	8244	12034	17057
62.5%	Data from NL	All	426	699	1220	2085	3433	5308	7821	10800	14258
62.5%	Data from NL	High	433	721	1208	2103	3496	5485	8109	11363	15046
62.5%	Data from NL	Medium	448	751	1324	2291	3847	6056	8989	12496	16500
62.5%	Data from NL	Low	435	744	1277	2222	3761	5950	8804	12361	16487
62.5%	Literature-E	All	561	1036	1948	3502	5829	9075	13144	18018	23206
62.5%	Literature-E	High	569	1053	1967	3528	5950	9293	13670	18828	24650
62.5%	Literature-E	Medium	582	1050	1957	3575	6018	9466	13897	19218	25111
62.5%	Literature-E	Low	579	1056	1981	3550	5973	9393	13914	19258	25038
62.5%	Literature-I_s	All	554	980	1807	3291	5642	9110	13716	19602	26308
62.5%	Literature-I_s	High	564	1014	1855	3344	5737	9399	14254	20426	27646
62.5%	Literature-I_s	Medium	557	1005	1861	3340	5764	9284	14242	20334	27638
62.5%	Literature-I_s	Low	547	996	1830	3320	5679	9256	14163	20272	27626
75.0%	Data from NL	All	534	980	1855	3358	5638	8778	12759	17281	22286
75.0%	Data from NL	High	539	983	1875	3367	5693	8996	13122	17933	23171
75.0%	Data from NL	Medium	560	1080	2035	3767	6365	9929	14452	19733	25435
75.0%	Data from NL	Low	557	1047	2010	3676	6263	9838	14352	19667	25424
75.0%	Literature-E	All	650	1235	2292	4148	6977	10716	15575	21199	27382
75.0%	Literature-E	High	649	1234	2329	4216	7108	11089	16109	22023	28709
75.0%	Literature-E	Medium	654	1230	2368	4263	7204	11254	16329	22471	29326
75.0%	Literature-E	Low	661	1242	2354	4263	7157	11192	16394	22555	29340
75.0%	Literature-I_s	All	729	1434	2900	5462	9436	14974	21998	30272	39108
75.0%	Literature-I_s	High	717	1479	2904	5504	9547	15297	22538	31211	40745
75.0%	Literature-I_s	Medium	728	1458	2894	5472	9534	15267	22476	31030	40602
75.0%	Literature-I_s	Low	730	1454	2909	5528	9603	15293	22512	31093	40564

Figure 1: **S3. Figure. The total number of deaths as simulation output for various parameter combinations.** The columns denote the different virus transmission probabilities and every row a certain combination of the probability of developing symptoms, the case fatality ratio and the possibility of developing immunity.

ICU.

Then, Figure 2 shows the MSE on the ICU for all possible parameter combinations, not only for the realistic ones.

Scenarios			Transmission probability								
Symptoms	CFR	Immunity	0.15	0.2	0.25	0.3	0.35	0.4	0.45	0.5	0.55
37.5%	Data from NL	All	1870	1524	902	245	195	2369	8900	10000	10000
37.5%	Data from NL	High	1857	1487	852	184	487	3805	10000	10000	10000
37.5%	Data from NL	Low	1865	1501	892	213	467	3829	10000	10000	10000
37.5%	Data from NL	Medium	1848	1470	902	216	469	3814	10000	10000	10000
37.5%	Literature-E	All	1970	1739	1276	679	165	399	2910	8419	10000
37.5%	Literature-E	High	1962	1669	1275	668	157	717	3758	10000	10000
37.5%	Literature-E	Low	1955	1725	1284	682	144	628	4161	10000	10000
37.5%	Literature-E	Medium	1943	1707	1273	646	159	680	3740	10000	10000
37.5%	Literature-I_s	All	2086	1996	1786	1475	978	451	230	1175	4040
37.5%	Literature-I_s	High	2089	1986	1796	1484	986	406	285	1431	5140
37.5%	Literature-I_s	Low	2097	1999	1828	1486	998	439	269	1454	5523
37.5%	Literature-I_s	Medium	2082	1991	1815	1451	959	404	290	1511	5375
50.0%	Data from NL	All	1643	1078	267	387	4542	10000	10000	10000	10000
50.0%	Data from NL	High	1639	1026	263	649	5303	10000	10000	10000	10000
50.0%	Data from NL	Low	1657	1008	218	872	6778	10000	10000	10000	10000
50.0%	Data from NL	Medium	1638	1019	222	783	6785	10000	10000	10000	10000
50.0%	Literature-E	All	1774	1319	540	154	2259	10000	10000	10000	10000
50.0%	Literature-E	High	1807	1336	509	220	2812	10000	10000	10000	10000
50.0%	Literature-E	Low	1782	1278	549	158	2723	10000	10000	10000	10000
50.0%	Literature-E	Medium	1776	1301	522	190	2573	10000	10000	10000	10000
50.0%	Literature-I_s	All	2002	1691	1164	445	292	2598	10000	10000	10000
50.0%	Literature-I_s	High	1965	1663	1125	437	351	3142	10000	10000	10000
50.0%	Literature-I_s	Low	1986	1720	1166	442	345	3097	10000	10000	10000
50.0%	Literature-I_s	Medium	1975	1693	1152	435	333	3243	10000	10000	10000
62.5%	Data from NL	All	1319	438	442	6485	10000	10000	10000	10000	10000
62.5%	Data from NL	High	1363	434	470	7315	10000	10000	10000	10000	10000
62.5%	Data from NL	Low	1306	377	664	8692	10000	10000	10000	10000	10000
62.5%	Data from NL	Medium	1342	422	741	9049	10000	10000	10000	10000	10000
62.5%	Literature-E	All	1446	591	268	4806	10000	10000	10000	10000	10000
62.5%	Literature-E	High	1512	640	270	5406	10000	10000	10000	10000	10000
62.5%	Literature-E	Low	1481	605	258	5735	10000	10000	10000	10000	10000
62.5%	Literature-E	Medium	1476	580	296	5351	10000	10000	10000	10000	10000
62.5%	Literature-I_s	All	1747	1184	350	792	7287	10000	10000	10000	10000
62.5%	Literature-I_s	High	1733	1166	305	941	8493	10000	10000	10000	10000
62.5%	Literature-I_s	Low	1756	1118	337	844	8096	10000	10000	10000	10000
62.5%	Literature-I_s	Medium	1739	1143	325	870	8224	10000	10000	10000	10000
75.0%	Data from NL	All	949	138	4722	10000	10000	10000	10000	10000	10000
75.0%	Data from NL	High	933	131	4827	10000	10000	10000	10000	10000	10000
75.0%	Data from NL	Low	879	157	6474	10000	10000	10000	10000	10000	10000
75.0%	Data from NL	Medium	887	154	6129	10000	10000	10000	10000	10000	10000
75.0%	Literature-E	All	1340	402	856	9807	10000	10000	10000	10000	10000
75.0%	Literature-E	High	1327	348	856	10000	10000	10000	10000	10000	10000
75.0%	Literature-E	Low	1323	367	954	10000	10000	10000	10000	10000	10000
75.0%	Literature-E	Medium	1281	385	868	10000	10000	10000	10000	10000	10000
75.0%	Literature-I_s	All	1389	429	931	10000	10000	10000	10000	10000	10000
75.0%	Literature-I_s	High	1415	393	989	10000	10000	10000	10000	10000	10000
75.0%	Literature-I_s	Low	1371	423	1002	10000	10000	10000	10000	10000	10000
75.0%	Literature-I_s	Medium	1405	442	954	10000	10000	10000	10000	10000	10000

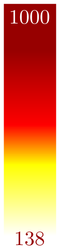

Figure 2: **S4 Figure. A heatmap representing the quality of prediction of various combinations of COVID-19 characteristics with respect to ICU occupation.** On the right a heatmap indicating that the darker the color is larger the MSE. The columns denote the different virus transmission probabilities and every row a certain combination of the probability of developing symptoms, the case fatality ratio and the possibility of developing immunity.

Percentage Infected and Immune individuals.

Figures 3 and 4 show the percentages of infected and immune individuals for all possible parameter combinations.

Scenarios			Transmission probability								
Symptoms	CFR	Immunity	0.15	0.2	0.25	0.3	0.35	0.4	0.45	0.5	0.55
37.5%	Data from NL	All	0.6	1.1	1.9	3.2	5	7.5	10.9	15	20
37.5%	Data from NL	High	0.6	1.1	2	3.3	5.2	7.9	11.4	15.9	21.5
37.5%	Data from NL	Medium	0.6	1.1	2	3.3	5.2	7.9	11.4	16	21.5
37.5%	Data from NL	Low	0.6	1.1	2	3.3	5.2	7.8	11.4	15.9	21.4
37.5%	Literature-E	All	0.4	0.8	1.3	2.1	3.3	4.9	7.1	9.9	13.4
37.5%	Literature-E	High	0.4	0.8	1.3	2.2	3.3	5	7.3	10.2	13.9
37.5%	Literature-E	Medium	0.4	0.8	1.3	2.1	3.3	5	7.3	10.2	13.9
37.5%	Literature-E	Low	0.4	0.8	1.3	2.2	3.3	5	7.3	10.2	14
37.5%	Literature-I-s	All	0.2	0.3	0.5	0.8	1.3	2	2.9	4.2	5.8
37.5%	Literature-I-s	High	0.2	0.3	0.5	0.8	1.3	2	2.9	4.1	5.8
37.5%	Literature-I-s	Medium	0.2	0.3	0.5	0.8	1.3	2	2.9	4.2	5.8
37.5%	Literature-I-s	Low	0.2	0.3	0.5	0.8	1.3	1.9	2.9	4.1	5.8
50.0%	Data from NL	All	0.7	1.3	2.3	3.8	6	9.1	13.1	18	23.9
50.0%	Data from NL	High	0.7	1.3	2.3	3.9	6.2	9.3	13.6	18.9	25.3
50.0%	Data from NL	Medium	0.7	1.3	2.4	4.1	6.6	10.1	14.7	20.4	27.3
50.0%	Data from NL	Low	0.7	1.3	2.4	4.1	6.6	10.1	14.7	20.5	27.3
50.0%	Literature-E	All	0.5	1	1.7	3	4.7	7.2	10.5	14.8	19.9
50.0%	Literature-E	High	0.5	1	1.8	3	4.8	7.4	10.8	15.3	20.8
50.0%	Literature-E	Medium	0.5	1	1.8	3	4.8	7.4	10.8	15.3	20.7
50.0%	Literature-E	Low	0.5	1	1.8	3	4.7	7.3	10.7	15.2	20.6
50.0%	Literature-I-s	All	0.2	0.5	0.9	1.5	2.5	3.9	5.8	8.5	11.8
50.0%	Literature-I-s	High	0.3	0.5	0.9	1.5	2.5	3.9	5.9	8.5	11.9
50.0%	Literature-I-s	Medium	0.3	0.5	0.9	1.5	2.5	3.9	5.9	8.6	12
50.0%	Literature-I-s	Low	0.3	0.5	0.9	1.5	2.5	3.9	5.9	8.6	12
62.5%	Data from NL	All	0.8	1.6	3	5.1	8.3	12.6	18.1	24.8	32.2
62.5%	Data from NL	High	0.8	1.6	3	5.1	8.3	12.8	18.6	25.5	33.5
62.5%	Data from NL	Medium	0.8	1.7	3.2	5.6	9.1	14	20.3	28	36.5
62.5%	Data from NL	Low	0.8	1.7	3.2	5.5	9	13.9	20.3	27.9	36.6
62.5%	Literature-E	All	0.6	1.3	2.5	4.5	7.3	11.4	16.7	23.1	30.5
62.5%	Literature-E	High	0.6	1.3	2.5	4.5	7.4	11.5	16.9	23.7	31.5
62.5%	Literature-E	Medium	0.6	1.3	2.5	4.5	7.4	11.6	17	23.8	31.7
62.5%	Literature-E	Low	0.6	1.3	2.5	4.5	7.4	11.5	17.1	23.9	31.8
62.5%	Literature-I-s	All	0.4	0.8	1.4	2.5	4.2	6.6	10	14.3	19.7
62.5%	Literature-I-s	High	0.4	0.8	1.5	2.6	4.3	6.7	10.1	14.6	20.1
62.5%	Literature-I-s	Medium	0.4	0.8	1.5	2.6	4.2	6.7	10	14.5	20
62.5%	Literature-I-s	Low	0.4	0.8	1.4	2.5	4.2	6.7	10	14.5	20
75.0%	Data from NL	All	0.9	2	3.8	6.7	10.9	16.7	23.8	32	40.7
75.0%	Data from NL	High	0.9	2	3.8	6.7	11	16.9	24.3	32.8	42.1
75.0%	Data from NL	Medium	1	2.1	4.1	7.3	12.1	18.6	26.6	35.9	45.7
75.0%	Data from NL	Low	1	2.1	4.1	7.4	12.1	18.7	26.7	36	45.9
75.0%	Literature-E	All	0.6	1.3	2.5	4.3	7.1	10.9	15.9	22.1	29.1
75.0%	Literature-E	High	0.6	1.3	2.5	4.4	7.2	11.1	16.2	22.6	30
75.0%	Literature-E	Medium	0.6	1.3	2.5	4.3	7.1	11	16.1	22.6	30.1
75.0%	Literature-E	Low	0.6	1.3	2.5	4.4	7.2	11.2	16.4	22.8	30.4
75.0%	Literature-I-s	All	0.5	1.2	2.3	4.1	6.9	10.9	16.3	22.8	30.4
75.0%	Literature-I-s	High	0.5	1.2	2.3	4.1	6.9	11	16.3	23.1	31
75.0%	Literature-I-s	Medium	0.5	1.2	2.3	4.1	6.9	10.9	16.2	22.9	30.8
75.0%	Literature-I-s	Low	0.5	1.2	2.3	4.1	7	11	16.4	23.1	30.9

Figure 3: **S5 Figure. The percentage of the population that was infected up to and including March 12 for various parameter combinations.** The columns denote the different virus transmission probabilities and every row a certain combination of the probability of developing symptoms, the case fatality ratio and the possibility of developing immunity.

Scenarios			Transmission probability								
Symptoms	CFR	Immunity	0.15	0.2	0.25	0.3	0.35	0.4	0.45	0.5	0.55
37.5%	Data from NL	All	0.8	1.3	2.1	3.4	5.2	7.7	11	15.2	20.1
37.5%	Data from NL	High	0.3	0.5	0.8	1.3	2	3	4.3	6	8.1
37.5%	Data from NL	Medium	0.1	0.2	0.4	0.6	1	1.5	2.2	3	4
37.5%	Data from NL	Low	0.1	0.1	0.2	0.3	0.5	0.7	1.1	1.5	2
37.5%	Literature-E	All	0.6	0.9	1.4	2.3	3.4	5.1	7.2	10	13.5
37.5%	Literature-E	High	0.2	0.3	0.5	0.9	1.3	1.9	2.7	3.9	5.2
37.5%	Literature-E	Medium	0.1	0.2	0.3	0.4	0.6	1	1.4	1.9	2.6
37.5%	Literature-E	Low	0.1	0.1	0.1	0.2	0.3	0.5	0.7	1	1.3
37.5%	Literature-I-s	All	0.2	0.3	0.6	0.9	1.3	2	2.9	4.2	5.8
37.5%	Literature-I-s	High	0.1	0.1	0.2	0.3	0.5	0.7	1.1	1.5	2.1
37.5%	Literature-I-s	Medium	0	0.1	0.1	0.2	0.2	0.4	0.5	0.8	1.1
37.5%	Literature-I-s	Low	0	0	0	0.1	0.1	0.2	0.3	0.4	0.5
50.0%	Data from NL	All	0.8	1.4	2.4	3.9	6.1	9.2	13.2	18.1	24
50.0%	Data from NL	High	0.4	0.7	1.2	2	3.1	4.7	6.8	9.5	12.6
50.0%	Data from NL	Medium	0.2	0.4	0.6	1.1	1.7	2.5	3.7	5.1	6.8
50.0%	Data from NL	Low	0.1	0.2	0.3	0.5	0.8	1.3	1.9	2.6	3.4
50.0%	Literature-E	All	0.6	1.1	1.8	3	4.8	7.3	10.6	14.8	19.9
50.0%	Literature-E	High	0.3	0.5	0.9	1.5	2.4	3.7	5.4	7.6	10.3
50.0%	Literature-E	Medium	0.3	0.5	0.9	1.5	2.4	3.7	5.4	7.6	10.3
50.0%	Literature-E	Low	0.3	0.5	0.9	1.5	2.4	3.6	5.3	7.5	10.2
50.0%	Literature-I-s	All	0.3	0.5	0.9	1.6	2.5	3.9	5.8	8.4	11.8
50.0%	Literature-I-s	High	0.1	0.3	0.5	0.8	1.3	2	2.9	4.2	5.9
50.0%	Literature-I-s	Medium	0.1	0.1	0.2	0.4	0.6	1	1.5	2.1	3
50.0%	Literature-I-s	Low	0	0.1	0.1	0.2	0.3	0.5	0.7	1.1	1.5
62.5%	Data from NL	All	0.9	1.7	3.1	5.2	8.4	12.6	18.2	24.8	32.2
62.5%	Data from NL	High	0.5	1	1.9	3.3	5.2	8	11.6	16	20.9
62.5%	Data from NL	Medium	0.3	0.6	1	1.8	2.9	4.4	6.4	8.7	11.4
62.5%	Data from NL	Low	0.1	0.3	0.5	0.9	1.4	2.2	3.2	4.4	5.7
62.5%	Literature-E	All	0.7	1.4	2.6	4.5	7.4	11.4	16.7	23.1	30.4
62.5%	Literature-E	High	0.4	0.9	1.6	2.8	4.6	7.2	10.5	14.7	19.6
62.5%	Literature-E	Medium	0.2	0.4	0.8	1.4	2.3	3.6	5.3	7.4	9.9
62.5%	Literature-E	Low	0.1	0.2	0.4	0.7	1.2	1.8	2.7	3.7	5
62.5%	Literature-I-s	All	0.4	0.8	1.5	2.6	4.2	6.6	10	14.2	19.5
62.5%	Literature-I-s	High	0.3	0.5	0.9	1.6	2.7	4.2	6.3	9	12.4
62.5%	Literature-I-s	Medium	0.3	0.5	0.9	1.6	2.6	4.1	6.2	8.9	12.3
62.5%	Literature-I-s	Low	0.3	0.5	0.9	1.6	2.6	4.1	6.2	8.9	12.3
75.0%	Data from NL	All	1	2	3.9	6.7	11	16.7	23.8	31.9	40.7
75.0%	Data from NL	High	0.7	1.5	2.9	5.1	8.3	12.6	18.2	24.5	31.4
75.0%	Data from NL	Medium	0.4	0.8	1.6	2.8	4.6	7	10	13.4	17.1
75.0%	Data from NL	Low	0.2	0.4	0.8	1.4	2.3	3.5	5	6.7	8.6
75.0%	Literature-E	All	0.7	1.4	2.5	4.4	7.1	10.9	15.9	22	28.9
75.0%	Literature-E	High	0.5	1	1.9	3.3	5.4	8.3	12.1	16.8	22.4
75.0%	Literature-E	Medium	0.3	0.5	0.9	1.6	2.7	4.1	6	8.4	11.2
75.0%	Literature-E	Low	0.1	0.3	0.5	0.8	1.4	2.1	3.1	4.3	5.7
75.0%	Literature-I-s	All	0.6	1.2	2.3	4.1	6.9	10.9	16.2	22.7	30.2
75.0%	Literature-I-s	High	0.4	0.9	1.7	3.1	5.2	8.2	12.2	17.2	23
75.0%	Literature-I-s	Medium	0.4	0.9	1.7	3.1	5.1	8.1	12	17	22.8
75.0%	Literature-I-s	Low	0.4	0.9	1.7	3.1	5.2	8.2	12.1	17.1	22.8

Figure 4: **S6 Figure. The percentage of immunity among the population based on individuals who were infected no later than March 12 for various parameter combinations.** The columns denote the different virus transmission probabilities and every row a certain combination of the probability of developing symptoms, the case fatality ratio and the possibility of developing immunity.
